# MPSI Manifestations and Treatment Outcome: Skeletal Focus

**DOI:** 10.3390/ijms231911168

**Published:** 2022-09-22

**Authors:** Giada De Ponti, Samantha Donsante, Marta Frigeni, Alice Pievani, Alessandro Corsi, Maria Ester Bernardo, Mara Riminucci, Marta Serafini

**Affiliations:** 1 San Raffaele Telethon Institute for Gene Therapy, IRCCS San Raffaele Scientific Institute, 20132 Milan, Italy; 2 Department of Molecular Medicine, Sapienza University, 00161 Rome, Italy; 3 Department of Pediatrics, Division of Medical Genetics and Metabolism, Zucker School of Medicine at Hofstra/Northwell, New York, NY 11021, USA; 4 Centro Ricerca M. Tettamanti, Department of Pediatrics, University of Milano-Bicocca, 20900 Monza, Italy; 5 Pediatric Immunohematology and Bone Marrow Transplantation Unit, San Raffaele Scientific Institute, 20132 Milan, Italy; 6 Pediatrics Department, Vita-Salute San Raffaele University, 20132 Milan, Italy

**Keywords:** lysosomal storage disease, mucopolysaccharidoses, mucopolysaccharidosis type I, lysosomal alpha-L-iduronidase, glycosaminoglycans, endochondral bone formation

## Abstract

Mucopolysaccharidosis type I (MPSI) (OMIM #252800) is an autosomal recessive disorder caused by pathogenic variants in the *IDUA* gene encoding for the lysosomal alpha-L-iduronidase enzyme. The deficiency of this enzyme causes systemic accumulation of glycosaminoglycans (GAGs). Although disease manifestations are typically not apparent at birth, they can present early in life, are progressive, and include a wide spectrum of phenotypic findings. Among these, the storage of GAGs within the lysosomes disrupts cell function and metabolism in the cartilage, thus impairing normal bone development and ossification. Skeletal manifestations of MPSI are often refractory to treatment and severely affect patients’ quality of life. This review discusses the pathological and molecular processes leading to impaired endochondral ossification in MPSI patients and the limitations of current therapeutic approaches. Understanding the underlying mechanisms responsible for the skeletal phenotype in MPSI patients is crucial, as it could lead to the development of new therapeutic strategies targeting the skeletal abnormalities of MPSI in the early stages of the disease.

## 1. Introduction

Mucopolysaccharidosis type I (MPSI) (OMIM #252800) is an autosomal recessive disorder affecting on average 1 in 100,000 live births, with females and males equally affected [1]. Increased birth incidence has been reported in North Europe, Saudi Arabia, Portugal, and Taiwan, with differences based on ethnic background and prevalence of common genetic variants [2,3]. MPSI is caused by biallelic pathogenic variants in the *IDUA* gene, encoding for the lysosomal alpha-L-iduronidase (IDUA) enzyme [4,5,6]. The deficiency of this enzyme causes accumulation of two types of glycosaminoglycans (GAGs), known as heparan sulfate (HS) and dermatan sulfate (DS). The storage of GAGs within the lysosomes disrupts cell function and metabolism, leading to a chronic and progressive multisystemic disease with a wide spectrum of phenotypic findings and disease severity. Historically, affected patients have been classified as having one of three MPSI syndromes, namely, Hurler syndrome (severe phenotype), Hurler–Scheie syndrome (moderate phenotype), and Scheie syndrome (mild phenotype) [7]. Patients are now described as having either severe MPSI or attenuated MPSI [8]. More than 350 causative variants in the *IDUA* gene have been identified to date, including missense, nonsense, splice site, and insertions, as well as small deletions and duplications [9]. Copy number variations have been also reported [10]. The most common causative variants reported worldwide include c.1205G > A (p.Trp402Ter), c.208C > T (p.Gln70Ter), c.1598C > G (p.Pro533Arg), and c.152G > A (p.Gly51Asp) [2,11,12]. c.1205G > A accounts for approximately 45% of disease alleles in the United States, whereas c.208C > T is mostly recorded in Russia and Scandinavia with a 50% frequency [2,13,14,15]. The c.1598C > G allele has the highest frequency in Morocco (92%), Tunisia (81%), and Algeria (54%) [16,17,18]. This variant has also been reported in the Mediterranean regions (42% of alleles) and Northern Europe [15]. The pathogen variant c.152G > A has the highest frequency in Norway (54%), Russia (42%), Poland (30%), and Austria (31%). The accurate prediction of genotype/phenotype correlation in MPSI has significant implications in order to determine the appropriate treatment and achieve optimal clinical outcomes. Patients homozygous or compound heterozygous for nonsense variants are predicted to have a more severe phenotype, compared to patients with some preserved enzymatic activity. Although there is a close genotype/phenotype correlation in MPSI, predicting the phenotype of patients with missense, splice site, and insertions, as well as small deletions and duplications, remains a challenge [4]. In addition, intrafamilial variability has been described in male siblings with MPSII and MPSIVA; thus, caution must be used when counseling families [19,20].

Since the majority of patients appear normal at birth, the diagnosis of MPSI is often delayed and not made until a few years of age. As a result, devastating and sometimes irreversible complications from the disease have already occurred [6,21,22].

**Figure 1:**
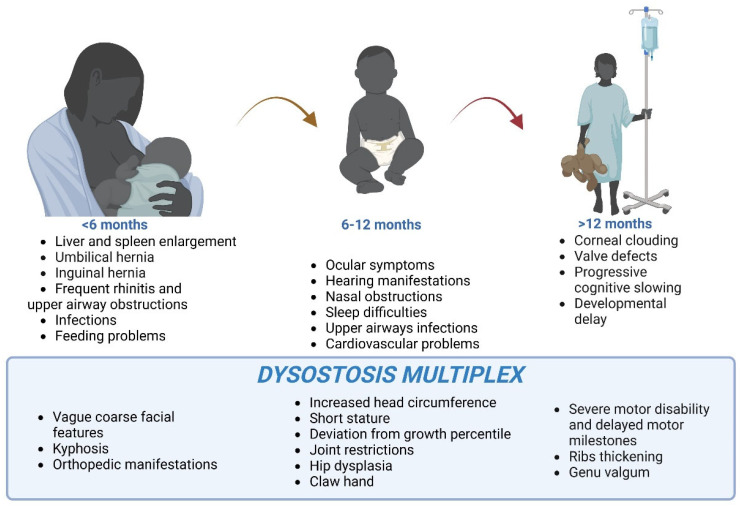
Disease manifestation onset in severe MPSI patients. Timeline of clinical manifestations in severe MPSI with focus on skeletal findings. Created with BioRender.com (accessed on 16 September 2022).

In some cases, coarse facial features, hepatosplenomegaly, abdominal and inguinal hernias, upper respiratory tract obstruction, frequent rhinitis, infections, and feeding difficulties can be apparent in the first few months of life (Figure 1) [21,23,24]. Ophthalmological and audiological abnormalities, as well as pulmonary and cardiovascular problems, can appear between 6 and 12 months of age [2,21,25,26,27,28]. In addition, patients with severe MPSI present progressive central nervous system (CNS) involvement that results in global developmental delays and intellectual disability [6,21,29,30,31]. If not diagnosed and treated, patients’ life expectancy is severely reduced, and survival is less than 10 years on average [32].

A prominent clinical manifestation of severe MPSI is *dysostosis multiplex*, a constellation of skeletal abnormalities. First described by Hunter in 1917, *dysostosis multiplex* includes a wide range of radiographical signs that are due to the abnormal formation of endochondral bone, such as genu valgum, coxa valga, thickened clavicles, shoe-shaped sella, oar-shaped ribs, flaring of iliac bones, and thoracolumbar kyphosis [7,29,33]. The progressive accumulation of GAGs in tendons, joints, and ligaments results in joint stiffness, contractures, and arthropathy, leading to motor dysfunction in the severe cases. The mechanisms responsible for *dysostosis multiplex* in MPSI patients are poorly understood, although it has been postulated that they are a direct consequence of altered endochondral ossification (see Section 3). Growth impairment is thought to be a direct consequence of GAG deposition in the epiphyseal plates [34], whereas macrocephaly has been postulated to result from the thickening of the calvarium, enlargement of cerebral structures, and accumulation of cerebrospinal fluid [35]. The aim of this review is to describe the pathological and molecular mechanisms responsible for the musculoskeletal impairment in MPSI patients. In addition, current and future therapeutic approaches are discussed, and their benefits and limitations reviewed.

## 2. Pathological Mechanisms Leading to GAG Accumulation

**Figure 2:**
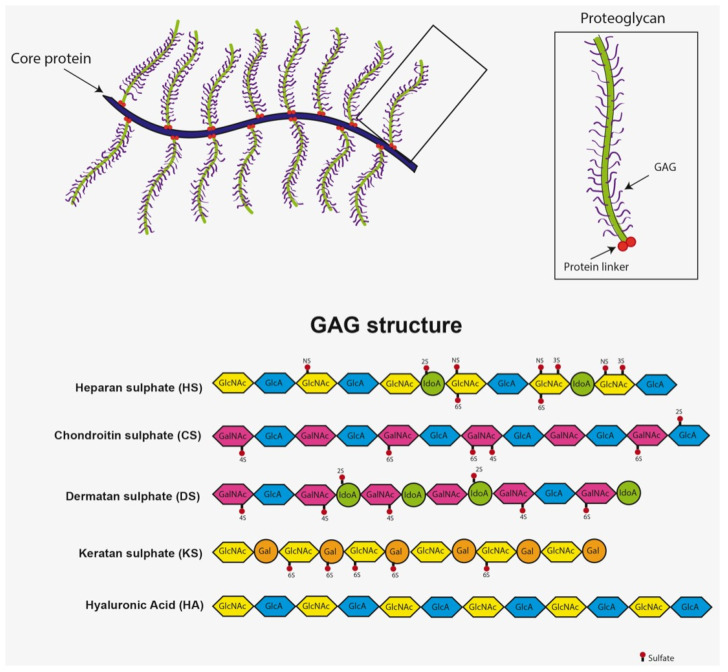
Schematic representation of a proteoglycan (**top**) and structure of principal GAG chains (**bottom**).

MPSI is characterized by a broad spectrum of phenotypic manifestations caused by GAG accumulation in different tissues. GAGs are sulfated polysaccharide chains that attach to core proteins, forming proteoglycans, that play critical physiological roles in various tissues, including cell signaling, stimulating growth and development, and extracellular matrix (ECM) hydration. The core proteins of proteoglycans can be transmembrane; therefore, GAGs can be a part of the ECM or part of the glycocalyx. GAGs include chondroitin sulfate, dermatan sulfate, heparan sulfate, keratan sulfate, and hyaluronic acid [36,37] (Figure 2).

The measurement of GAGs in urine and blood represents a valid method for diagnosis and, potentially, for prognosis and/or monitoring therapy [38,39,40]. Liquid chromatography tandem mass spectrometry (LC-MS/MS) methods are considered more accurate than dye-spectrometric and thin-layer chromatography techniques, as they can quantify individual classes of GAG-derived disaccharides from various tissues, including cerebrospinal fluid and dried blood spots [38]. Patients’ age and development, type of GAGs analyzed, phenotype severity, and technique used need to be taken into account as potential causes for false-negative results [41]. The measurement of GAGs by LC-MS/MS has also been shown to be a valid biomarker for newborn screening [39]. There is still no consensus on what method is the gold standard for disease monitoring, although the use of disease-specific, non-reducing end carbohydrate biomarkers has shown promising results [42].

Beyond their classically recognized structural function, GAGs actively interact as co-receptors with different growth factors (GFs) and adhesion molecules, regulating cell proliferation and adhesion. They also interact with tyrosine kinases and toll-like receptors, modulating many biochemical processes involved in bone remodeling, angiogenesis, and wound healing [43,44,45].

GAGs have a mechanical role in ECM that is important for the structure of dynamic tissues, such as tendons, cartilage, blood vessels, and brain [37,46]. It has been demonstrated that GAGs take part in regenerative processes. In addition, HS-proteoglycans are known as regulators of the adaptive immune system, modulating the activity of dendritic cells, macrophages, and B lymphocytes, as well as cytokine production [47]. In particular, it has been demonstrated that HS binding to toll-like receptor 4 (TLR4) promotes pathways involved in T cell growth, antigen-presenting cell function, and co-stimulatory molecule upregulation [47,48,49]. Activation of the adaptive immune response plays a role in the pathogenesis of MPSs, aggravating an already impaired cellular homeostasis.

GAG accumulation inside cells in ECM and tissues causes increased water absorption, abnormal deposition of collagen fibers, and augmented cell size [46]. Overall, the most prominent clinical manifestations of MPSI involve the skeletal, cardiovascular, visual, integumentary, and nervous systems. This tropism can be considered as a direct consequence of DS and HS deposition in MPSI patients. DS is present in corneas, kidneys, and epithelium, as well as muscular and skeletal tissues, and contributes to their growth [50]. Similarly, HS is located in the basement ECM structure or it is secreted, where it modulates intra- and extracellular connections and interacts with several molecules based on their sulfation profile and charge [51].

GAGs, alongside amino acids, sphingolipids, and glycogen, are metabolized inside the lysosomes through the action of several enzymes. Lysosomes play a crucial role in macromolecules’ degradation and trafficking, as well as in cell homeostasis through their interaction with other organelles [52,53,54,55]. In case of an enzymatic block, GAGs accumulate inside the lysosomes, impairing their function.

Lysosomal dysfunction severely affects the musculoskeletal system in patients with MPSI. In fact, in order to properly function, osteoclasts rely on lysosomes, as they require both proteases and pH acidification for bone resorption [56]. Lysosomes are fundamental for vesicular trafficking, as well as cathepsin K and TRAP activity. In addition, lysosomes are thought to be crucial for osteoblasts’ proper differentiation, as demonstrated by reduced ER stress and overall increased lysosome presence observed during this process [57,58]. In addition, bone mineralization highly depends on calcium phosphate transport inside matrix vesicles, a process that is carried out by lysosomes inside osteoblasts [58].

**Figure 3:**
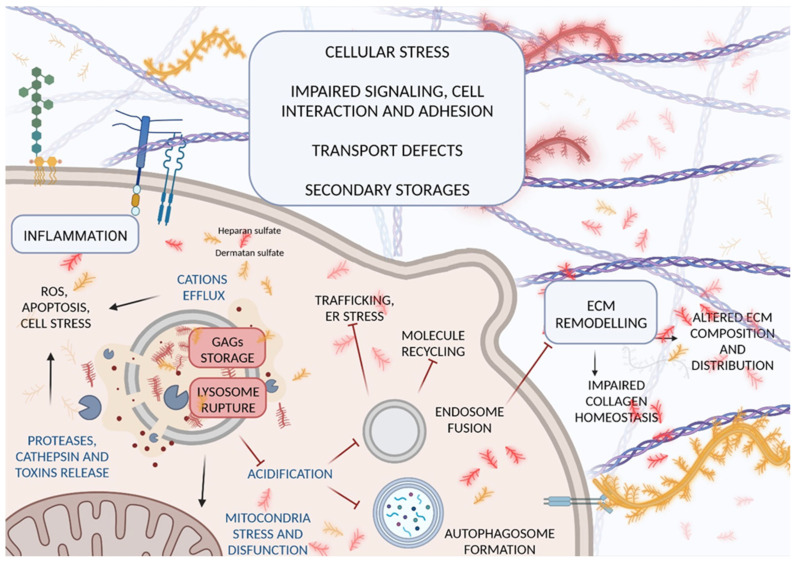
Representative pathological MPSI cascade. GAG accumulation inside the lysosome could be responsible for lysosome rupture with subsequent release of proteases, cathepsins, and toxic products inside the cytoplasm. This could potentially result in mitochondrial oxidative stress, ROS formation, impaired cell function, and eventually, apoptosis. Created with BioRender.com (accessed on 16 September 2022).

Lysosomes’ multi-functional roles could explain the broad spectrum of phenotypic manifestations in patients with lysosomal storage disorders following GAG deposition. Alteration of physiological pH, proteases’ impairment and their release inside the cell, and apoptosis activation cause a block in cellular trafficking, molecule recycling, and endosome autophagy, and trigger the TLR4 signaling pathway with subsequent tissue inflammation, oxidative stress, and eventually, cell death [59,60,61,62,63]. Secondary storage of toxic substances has been observed in many MPSs, both inside the lysosomes and in other cell compartments, and it is hypothesized to be responsible for nervous system involvement in MPSI (Figure 3) [64,65].

## 3. Endochondral Bone Formation

Bone formation follows two possible mechanisms: intramembranous ossification, in which bone develops directly from mesenchymal stromal cells (MSCs), such as in cranial and facial bones, and endochondral ossification, which involves the replacement of a hyaline cartilage template with bony tissue.

Chondrocytes play a major role in endochondral ossification. This process begins during fetal life, about 6 to 8 weeks after conception, with the migration and the condensation of MSCs to the sites of future skeletogenesis. Aggregated MSCs, located in the central area of condensation, differentiate into chondrocytes and generate a cartilaginous anlagen that will be resorbed and replaced by bone and bone marrow (BM) [66].

MSCs located in the outermost area of condensation become perichondral cells and then osteoblastic cells, generating the primitive bony collar. Chondrocytes secrete a matrix that allows cartilage mold to expand and grow, as well as differentiate into hypertrophic and terminal chondrocytes, while the cartilaginous matrix undergoes mineralization [67]. Terminal chondrocytes follow two different fates: they can become apoptotic or they can differentiate into osteoblasts, contributing to trabecular bone formation [67,68]. Blood vessels invade the hypertrophic cartilage through the bony collar, carrying nutrients, osteoprogenitor cells, and osteoclast progenitors. While osteoclasts reabsorb the hypertrophic cartilage, osteoprogenitor cells differentiate into osteoblasts and begin bone deposition on the cartilage matrix remnants. This gives rise to the primary ossification center (POC), in which the first trabecular bone is formed [69]. Meanwhile, cartilage continues to grow at both ends of the long bones (the future epiphyses), in which the same sequence of events will occur, giving rise to the secondary ossification center (SOC) [70]. Elongation of each endochondrally formed bone occurs at the growth plates, which are highly specialized cartilage regions physically placed at the extremities of the POC, and then between the POC and SOC. At these sites, chondrocyte differentiation stages (proliferative, pre-hypertrophic, hypertrophic, and terminal) occur in highly defined regions, generating the different layers that characterize the growth plate. The orderly sequence of chondrocyte proliferation, ECM secretion, and chondrocyte hypertrophy within the growth plate is crucial in order to allow bone elongation. This process is highly regulated by complex intra- and extracellular signaling pathways.

### 3.1. Principal Transcription Factors and Signaling Pathways in Endochondral Bone Formation

Chondrocytes, at the various differentiation stages within the growth plate, are characterized by specific and distinct gene expression profiles. Both immature chondrocytes (resting chondrocytes) and proliferative chondrocytes express the key SRY-box transcription factor 9 (SOX9), along with SOX5 and SOX6 [71]. These transcription factors activate chondrocyte differentiation through the expression of specific genes, such as the ones encoding for collagen type II alpha 1 (*Col2a1*) and aggrecan (*Acan*) [72]. At the pre-hypertrophic stage, chondrocytes silence the expression of *Col2a1* and *Acan*, and upregulate the collagen type X alpha 1 chain (*Col10a1*), Indian hedgehog (*Ihh*), parathyroid hormone receptor 1 (*Pthr1*), and runt-related transcription factor 2 (*Runx2*) [73].

**Figure 4:**
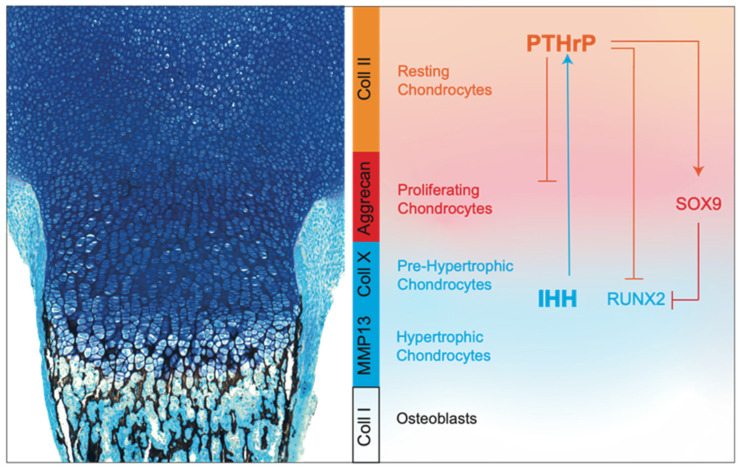
Main transcription factors and signaling pathways in endochondral bone formation. The left panel shows an undecalcified von Kossa/methylene blue-stained section of the femur of 1-day-old mouse showing mineralized matrix (black color) in the hypertrophic zone and in the underlying trabecular bone. The right panel illustrates the main matrix proteins and molecules secreted by chondrocytes and their transcription factors’ expression during the different differentiation stages. Resting and proliferating chondrocytes express SOX9 and produce collagen type II and aggrecan, respectively; pre-hypertrophic and hypertrophic chondrocytes express IHH, which induces the expression of PTHrP close to the articular region. PTHrP, in turn, blocks the expression of IHH in lower chondrocytes and regulates chondrocyte proliferation and hypertrophy. In addition, PTHrP maintains chondrocytes in their proliferative phase, promoting the activity of SOX9 and the inhibition of RUNX2, which is expressed by pre- and hypertrophic chondrocytes, and it is important for the production of collagen type X and MMP13 and the induction of osteoblastogenesis.

Hypertrophic chondrocytes continue to produce COL10A1, as well as RUNX2, and activate the expression of vascular endothelial growth factor A (*Vegfa*). In contrast, terminal chondrocytes silence genes encoding for collagen proteins and express matrix metalloproteinase 13 (*Mmp13*), together with *Vegfa* [74]. These genes are important for angiogenesis, which ultimately promotes the recruitment of osteoclasts and osteoblasts, essential for the production of trabecular bone (Figure 4).

- SOX9. The transcription factor SOX9 has a pivotal role in chondrocyte differentiation and cartilage formation [75,76]. In columnar chondrocytes, SOX9 is important to maintain cell proliferation and to directly silence genes that are normally expressed by hypertrophic chondrocytes, such as *Col10a1* and *Vegfa* [77,78]. In contrast, in early pre-hypertrophic chondrocytes, it directly and indirectly activates *Col10a1*, thus promoting the initial step of hypertrophic differentiation [79]. Finally, SOX9 is downregulated in hypertrophic chondrocytes as a necessary step for vascular invasion and subsequent bone deposition.
  SOX9 regulates growth plate development and chondrocyte differentiation through multiple mechanisms. For example, SOX9 is able to inhibit WNT signaling, whose members, in particular WNT5A and WNT5B, are known to be important for the progression of chondrocytes to hypertrophic maturation [80]. SOX9 modulates this pathway by interacting directly with βcatenin and promoting its nuclear translocation and degradation by the ubiquitination 26/S proteasome pathway [81,82]. In addition, SOX9 modulates RUNX2, another key transcription factor that directs both chondrocyte maturation and osteogenic differentiation during skeletal development [83]. SOX9 promotes RUNX2 degradation in a proteasome-independent, but phosphorylation-dependent, manner, and the balance between these two transcription factors is critical for correct growth plate formation (Figure 4) [84,85].
- IHH. IHH is essential for embryonic skeletal development, as well to modulate postnatal skeletal growth [86,87]. In the growth plate, IHH is expressed in the hypertrophic zone and regulates both proliferation and differentiation of chondrocytes [86]. Mice with global *Ihh* knockout show abnormalities in long bones, which are shorter compared to those of wild-type mice due to a marked reduction of chondrocyte proliferation with thinning of the cartilage region. In addition, they display a premature chondrocyte hypertrophy and absence of mature osteoblasts [86]. IHH coordinates events in the endochondral ossification via parathyroid hormone-related protein (PTHrP)-independent or -dependent pathways. In fact, similarly to *Ihh^−/−^* mice, transgenic models with the cartilage-specific ablation of *Smo*, which encodes for a G protein-coupled receptor that transduces the hedgehog’s protein signal, exhibit reduced chondrocyte proliferation, indicating that this process requires a direct IHH input [87]. However, in the same mice, chondrocyte differentiation and maturation appear similarly as in wild-type mice, suggesting that IHH does not regulate directly chondrocyte hypertrophy, but is instead dependent on its action on the PTHrP pathway [87].
- PTHrP. PTHrP is required for the maintenance of chondrocytes in their proliferative phase and to delay the hypertrophic one [88,89]. PTHrP establishes a feedback loop with IHH that is necessary to regulate chondrocyte proliferation and hypertrophy. Specifically, IHH, expressed by hypertrophic chondrocytes, stimulates the chondrocytes close to the articular region to produce PTHrP, which inhibits IHH expression in lower chondrocytes, keeping them proliferating. When chondrocytes are no longer reached by PTHrP, they stop to proliferate and start to secrete IHH, thus closing the loop [90,91,92,93,94].
  *Pthrp^−/−^* mice exhibit a reduction in the height of the proliferative zone, whereas overexpression of *Pthrp,* specifically in chondrocytes, results in delayed endochondral ossification and chondrodysplasia [95]. Interestingly, it has been shown that the proliferation rate of chondrocytes in *Pthrp^−/−^* mice is not affected. This indicates that the depletion of the proliferative zone results from an accelerated rate of chondrocyte differentiation, rather than from a change in the proliferative activity [96]. This is in agreement with the inhibitory function of PTHrP on two transcription factors involved in chondrocyte hypertrophy, such as the myocyte enhancer factor-2 and RUNX2 [97,98]. However, Huang et al. have suggested that PTHrP signaling retains chondrocytes in a proliferative phase also by inducing a PKA-mediated phosphorylation and activation of SOX9 (Figure 4) [99].
- FGFs. The fibroblast growth factor (FGF) protein family consists of 23 members, which have different roles in various biological processes. FGFs bind and activate four receptor tyrosine kinase molecules (FGF receptors, FGFRs), which have a specific and distinct expression pattern in the growth plate. *Fgfr1* and *Fgfr2* are both expressed in the perichondrium and periosteum; conversely, *Fgfr3* is expressed predominately in proliferating chondrocytes and in pre-hypertrophic chondrocytes [100].
  Among the four receptors, the function of FGFR3 is the most well understood. Ablation of *Fgfr3* in mice causes the expansion of proliferating and hypertrophic chondrocytes, whereas in humans, loss-of-function pathogenic variants in the *FGFR3* gene could cause the camptodactyly, tall stature, scoliosis, and hearing loss (CATSHL) syndrome [101,102,103]. By contrast, the overexpression of *Fgfr3* in mice and gain-of-function pathogenic variants in humans decrease the chondrocyte proliferation rate, leading to the development of achondroplasia [104].
  *Fgfr3* is known to inhibit chondrocyte proliferation through increased expression of *Snail1*, that activates the signal transducer and activator of the transcription 1 (STAT1) pathway [105,106]. FGFR3-induced chondrocyte growth arrest is also dependent on the upregulation of cell-cycle inhibitors, such as p21 and p27, and requires the activated (phosphorylated) form of p107 and p130, but not pRb [107,108]. On the other side, conflicting data have been reported regarding its effect on chondrocyte maturation. Murakami et al. demonstrated that FGFR3 delays chondrocyte maturation through the MAP kinase (MAPK) pathway. In contrast, Minina and colleagues proved that, in mouse embryonic limb explants, FGF signaling acts upstream of the IHH/PTHrP and accelerates late steps of chondrocyte hypertrophy [109]. Furthermore, subsequent work by Dailey et al. demonstrated that the FGF treatment of rat chondrosarcoma chondrocytes modulates the gene expression profile to favor chondrogenic hypertrophy [110]. Altogether, these data suggest that FGF inhibits proliferation and the initial stage of hypertrophy, but then promotes maturation into late hypertrophic chondrocytes.

## 4. Bone Alterations in Mucopolysaccharidoses: From Patients to Animal Models

Patients affected by MPSI show impaired bone formation with delayed growth, reduced bone mineral density, and ultimately, the development of osteopenia and osteoporosis [111,112]. Studies conducted on patients with severe MPSI revealed irregularities in chondrocyte organization and maturation within the growth plate, but the underlying mechanisms responsible for their skeletal phenotype are poorly understood [113]. In recent years, different types of animal models, including transgenic and inbred strains, have been used to investigate MPSs. However, studies on molecular mechanisms that specifically concern MPSI are still very few. For this reason, in this review we report the most important findings for all types of MPSs; they could be relevant for the understanding of the MPSI skeletal phenotype, based on the similarities observed in this group of diseases.

- **Skeletal Alterations**. MPSI and MPSII mice, as well as MPSI cats and dogs, present musculoskeletal deformities similar to those detected in affected patients [114,115,116,117]. In particular, they showed facial dysmorphisms, coxofemoral subluxation, fusion of the cervical vertebrae, pectus excavatum, and thickening of vertebrae arches. Shortened bones (femurs, tibiae, and lumbar vertebrae), a cardinal feature of human disease, were not observed in these models, in which bone lengths appeared similar to those of wild types [116,118]. In contrast, a reduction in bone length was detected in MPSVI rats and cats and in MPSVII dogs [119,120]. The MPSI and MPSVII canine models displayed lower bone mineral density compared to controls, similarly to what is observed in MPSI patients [121,122]. Conversely, mice models of MPSI and MPSVII showed higher bone mineral density, compared to their age-matched wild-type counterparts [118]. In particular, MPSI mice developed a progressive high bone mass phenotype, with a significantly reduced number of both osteoclasts and osteoblasts. Kuehn et al. investigated the activity of osteoclasts and their role in this high bone mass phenotype. Specifically, the authors demonstrated that BM transplantation from MPSI donor mice into wild-type recipient mice did not reproduce a high bone mass phenotype. Thus, they concluded that the increase in the trabecular bone could be due to impaired bone remodeling, determined by GAG accumulation in the bone matrix, rather than to defective osteoclast function [123].
- **Growth plate morphological abnormalities**. Both inbred strains and transgenic MPS animal models were able to also reproduce the abnormal growth plate morphology observed in the human disease, specifically, the disorganization of the columnar architecture in the proliferating and hypertrophic zones. Histological analysis of femurs of MPSI mice revealed thickening of the growth plate, in which the proliferative zone was thin and presented a disorganized distribution of chondrocytes [124,125]. In contrast, the hypertrophic zone was wider compared to healthy mice and showed an increased amount of large and swollen hypertrophic chondrocytes. Furthermore, it revealed that cartilage was retained within the mineralized cortical bone, and that the latter had lost its lamellar structure and organization [118,124].
  Abnormalities in chondrocytes, with enlarged lysosomes and growth plate disorganization, were also observed in MPSI dog and cat models, MPSVI cat models, and MPSIIIA, MPSIVA, and MPSVII mouse models [126,127,128]. In MPSVII dogs, lysosomes filled with storage materials were also detected in osteoblasts, osteoclasts, and osteocytes [129].
- **Molecular consequences of GAG accumulation**. GAGs are involved in multiple cellular pathways, through the regulation of the GF function and matrix release of cytokines and chemokines [130]. In addition, GAGs bind GF receptors, promoting conformational changes of the receptors themselves or of their ligands. Proteoglycans of the HS family can bind to a variety of growth factors, including members of the FGF family, bone morphogenic proteins (BMPs), transforming growth factor β (TGFβ), and WNT signaling pathways (reviewed in Bernfield et al.) [131]. It has been shown that HS-glycosaminoglycan is necessary in order for FGF ligands to bind to their cognate receptors [132]. However, the GAG excess in cells from MPSI patients results in the impairment of FGF/FGFR binding, and the FGF pathway activity was affected at an early stage of development and before the onset of bone defect in zebrafish and mice MPSII models [133,134]. GAG accumulation also impaired BMP-4 signaling in human multipotent adult progenitor cells derived from MPSI patients [135].
  Accumulation of HS in MPS patients does not occur in lysosomes only, but also affects other cellular and extracellular compartments. In MPSI and in MPSIIIB fibroblasts, extracellular accumulation of HS resulted in excessive binding of FGF2 and impaired activity of the growth factor, which was restored by reducing the excess HS [37].
  GAG affinity to growth factors is determined by their sulfation pattern [125]. Not only is the undegraded GAG accumulation involved in the pathogenesis of severe MPSI, but the abnormal GAG structure and sulfation pattern are involved as well. It has been shown in liver and brain tissues from 12-week-old MPSI mice that HS chains contain significantly increased N-, 2-O-, and 6-O-sulfation [136]. By contrast, cultured human BM-derived multipotent adult progenitor cells isolated from MPSI patients showed a reduction in 6-O sulfation of HS. This process results in impaired FGF-2 activity, as demonstrated by the fact that the replacement of normal HS on MPSI cells’ surfaces restored its function. The reduction in 6-O sulfation of HS in MPSI patients contributes to impaired FGF-2 activity, as demonstrated by the fact that the replacement of normal HS on MPSI cells’ surfaces restores its function [133]. In addition, it has been shown that GAG accumulation in MPSs could lead to growth plate anomalies, altering different processes involved in endochondral ossification, in particular, chondrocyte proliferation, as well as POC and SOC formation.
  How GAG accumulation in chondrocytes affects their proliferation capacity remains largely unknown. It has been shown that the growth plate of MPSVII mice exhibited a decreased chondrocyte proliferation, caused by increased activity of the anti-proliferative STAT1 and decreased activity of the pro-proliferative STAT3 factors [129,137]. In particular, STAT3 showed a markedly reduced phosphorylation at tyrosine705 as compared to normal mice, probably as the result of the diminished levels of leukemia inhibitory factor and IHH [137]. Recently, it has been shown that MPSVII chondrocytes are able to enter the cell cycle, but their ability to complete the cell cycle is impaired, thus leading to a delay in cellular proliferation and differentiation [129]. In MPSVII murine and canine models, abnormalities of chondrocytes were shown to be associated with a delayed formation of POC and SOC [138,139]. To explain this finding, it has been postulated that GAG accumulation in chondrocytes could alter the cellular processes involved in the transition of cartilage to bone, namely, hypertrophy, cartilage resorption, angiogenesis, and osteogenic differentiation. Peck et al. demonstrated the persistent expression of SOX9 in MPSVII dogs [139]. Since SOX9 directly represses the production of COL10A1, VEGF, and RUNX2 (see Section 3.1), its persistent expression could be responsible for the altered chondrocyte hypertrophy and the delayed bone formation. Indeed, the molecular profiling of epiphyseal tissues, isolated from 14-day-old MPSVII dogs, presented the downregulation of *COL10A1*, as well as *RUNX2* and other genes important for osteogenic differentiation, such as alkaline phosphatase (*ALPL*), osteocalcin (*BGLAP*), and osteopontin (*SPP1*). In addition, MMP, WNT/beta catenin, and BMP pathways exhibited significantly altered overall expression [140]. MMPs are enzymes involved in cartilage degradation and are responsible for bone remodeling and angiogenesis. MMP13 is expressed in hypertrophic chondrocytes and, together with MMP9, facilitates angiogenesis and, subsequently, the migration of bone-forming cells, allowing the formation of POC and SOC [140]. Downregulation of *Mmp13* has been observed also in MPSI mice [137]. Interestingly, MMP13 transcription is controlled by RUNX2, and therefore, it may be possible that the MMP13 downregulation in MPS chondrocytes could be an indirect consequence of persistent SOX9 expression [141].
  Finally, it has been demonstrated that MPSI mouse bones have an altered cathepsin K activity. Cathepsin K is a cysteine protease member of the cathepsin lysosomal protease family. It is highly expressed in osteoclasts, and it is responsible for collagen type II degradation. In MPSI mice, HS and DS accumulation blocks the catabolic activity of cathepsin K, thus resulting in a decreased cartilage resorption that contributes to the growth plate pathology of MPSI [142].

## 5. Therapeutic Options for MPSI and Focus on Bone Limitations

Over the past few decades, disease-specific therapies have been developed thanks to in-depth studies conducted in animal models and, together with supportive treatments, have improved patients’ quality of life. Because of the clinical heterogeneity of MPSI onset, the degree of disease progression, growth potential, and clinical phenotype should be considered when determining the appropriate treatment strategy, especially since these factors impact treatment effectiveness. Because MPSI symptoms worsen with time and a therapeutic delay could aggravate the patients’ medical conditions, early diagnosis and treatment are crucial and require a multidisciplinary team [21].

Many treatment strategies developed in the last decades rely on the cross-correction mechanism, firstly described by Fratantoni and Neufeld [143]. This exploits the ability to reintroduce into the endosomal system lysosomal proteins that have reached the extracellular compartment through the mannose-6-phospate (M6P) tag, and to correctly enter the lysosomes [144,145]. This cross-correction principle could be exploited by affected cells that lack the IDUA enzyme for recovering the enzymatic activity; in particular, these released hydrolases can be similarly uptaken by binding to M6P, since these cells possess the M6PR on their surface. The main therapeutic approaches for treating MPSI patients include direct intravenous injection of the human recombinant IDUA enzyme (enzyme replacement therapy, ERT), transplantation of donor-derived hematopoietic stem cells (HSCs) or autologous gene-modified cells with the ability to release the defective enzyme, or direct modification of the recipient cells by in vivo gene therapy.

Before these therapeutic strategies were introduced, the only treatment option for affected patients included the symptomatic treatment of disease-related complications. These include interventions that had the purpose of reducing secondary complications, including orthopedic, corneal, and cardiac surgeries, adenoidectomy, and median nerve-releasing procedures, among others [6,146]. These interventions carry a high risk of complications for affected patients, related to anesthesia and intubation [6,147,148].

Children diagnosed with MPSs may have discrepancies in their gross and fine motor abilities, and should be referred to a physical therapist as soon as possible after diagnosis [149]. Physical therapy can be beneficial to limit the impact changes in the body structures have on functional abilities. More importantly, physical therapy can facilitate independence and participation in age-appropriate activities. Functional skills such as moving between positions or completing age-appropriate daily routines should be emphasized [149]. Management of kyphosis/scoliosis through strengthening activities is especially important. Although it is hard to prevent the progression of kyphoscoliosis, physical therapy can delay the postural collapse biomechanically that causes secondary functional impairment (such as restrictive pulmonary disease and reduced cardiopulmonary performance) and reduce pain [150,151]. Children with decreased chest expansion may benefit from retraining of the inspiratory muscles to decrease episodes of dyspnea. Physical therapy, such as Swedish massage, posturing, and passive and active mobilization, could be applied for regaining hip mobility/full range of motion, strengthening the hypotonic muscles, rebalancing the pelvic asymmetry, and increasing the passive and active stability of hip joints [152]. Adults are more likely to have developed several complications. Hence, treatment in adults is carried out to maintain improvement, slow disease progression, and make the best use of preserved functions [149].

### 5.1. Hematopoietic Stem Cell Transplantation

Allogeneic hematopoietic stem cell transplantation (HSCT) is considered the standard of care for severe MPSI with CNS involvement, but with preserved neurocognitive function (intelligence quotient, IQ ≥ 70), below the age of 2.5 years [153]. HSCT represents a valid strategy for many lysosomal storage disorders because of the repopulation and differentiation of normal donor cells that can release functional enzymes for affected cells. Since injected hematopoietic stem cells can spread to different tissues, even non-hematopoietic ones, and differentiate into tissue-resident macrophages in the CNS, lungs, and liver, a long-lasting therapeutic effect can be achieved [144,145]. The enzymatic correction could be provided by the IDUA release from leukocytes and spread to other peripheral compartments through lymphatic and blood circulation [154].

Since the first HSCT on a MPSI patient in 1981, HSCT has significantly improved many MPSI symptoms and patients’ quality of life. For example, cardiopulmonary symptoms, a frequent cause of death in affected patients, have significantly improved along with survival rates. HSCT promoted a reduction in GAG storage, with subsequent improvement in hepatosplenomegaly, pulmonary and myocardial alterations, facial appearance, growth, and auditory ability [155,156,157,158]. Furthermore, HSCT was also found to prevent the progression of neurocognitive decline in affected patients, especially when treatment was started early in life [159,160,161,162]. In regard to the effects of HSCT on *dysostosis multiplex*, after HSCT, MPSI patients demonstrated improvements in joint stiffness and range of motion [154,155,158,163]. Yasuda et al. also reported normal chondrocytes and the hyaline matrix in the spine and L2 disc of affected patients, although some degree of ECM disorganization in collagen disposition and lacunar enlargement was still present [164].

Despite an overall reduction in disease burden and progression, HSCT presents some limitations [155]. In particular, HSCT is not able to reverse damage that has already occurred, such as to nervous system involvement, cardiac valve and ocular abnormalities, and also, skeletal alterations. The limitations of HSCT treatment have been postulated to be at least partially due to poor enzyme diffusion into these anatomical compartments [157,161,165,166]. Field et al. observed the persistence of intracellular undegraded GAGs after HSCT in a patient’s flexor retinaculum biopsy, suggesting a limitation of the treatment effect on skeletal manifestations [166]. Moreover, the benefits of HSCT were only temporary in some patients, with a reoccurrence of disease progression years after treatment [144,156,158,166,167].

The limitations of HSCT are especially apparent on the skeletal disease burden. For this reason, even after HSCT, patients still had to undergo surgical procedures in order to correct spinal deformities (scoliosis and kyphosis), hip dysplasia, median nerve compression, joints stiffness, hand and foot limitations in the range of motion, and genu valgum [144,156,158,166,168]. In almost all cases, further musculoskeletal deterioration occurred, requiring additional surgical intervention or aftercare for correcting and improving residual symptoms [146,166,167,169]. In addition to these skeletal manifestations, persistence of cardiac deformities, neurological involvement, and airway obstructions worsened the clinical outcome. Treatment maintenance after surgical procedures is tailored based on the severity of the skeletal deformities, as well as the patients’ age, and has the goal of increasing and preserving joint motility [169]. Residual kyphoscoliosis, usually in the lumbar region, is first addressed with an orthosis application for ameliorating the walking ability [146]. Moreover, since hip dysplasia persistence could be responsible for luxation, it is surgically treated for stabilization. HSCT’s limited effects on spinal alterations could be due to a combination of delayed treatment and the irreversibility of GAG deposition. The progression of spinal compression, caused by vertebral bodies’ ossification defects, often leads to nerve compression with subsequent neurological manifestations. Foot and ankle orthosis, hemiepiphysiodesis, and osteotomy of long bones are often performed as surgical interventions after HSCT, but they can lead to complications, such as mobility reduction and implant requirements [170,171,172]. There is still no consensus on what could be the best treatment strategy (bracing over surgery) or treatment timeline for children. Spinal growth could reduce the procedure severity, but higher risks due to comorbidities need to be taken into account [146]. Moreover, general anesthesia requires MPSI patients to be closely monitored, as they are at a high risk for complications, including cerebral lesions during the procedure. This is due to kyphosis, reduced spine mobility, macroglossia, and limited jaw opening [173].

Although HSCT fails to restore a normal growth pattern, transplanted MPSI patients presented a remarkable height gain compared to untreated gender- and age-matched individuals [174]. A trial of recombinant human growth hormone may be appropriate in children with MPSI or II that present with severe short stature and growth failure. However, the efficacy of therapy should be evaluated after 1 year, and discontinued if no increase in the growth velocity or height standard deviation score are recorded [175]. In general, the response to therapy is highly variable among different individuals, and could be affected by conditioning regimens pre-HSCT, as well as cell source and time of intervention [175,176,177,178,179,180].

Together with MPSI clinical severity, several factors take part in the HSCT outcome, such as patients’ age at the time of intervention (better outcomes are observed with early treatment); the conditioning regimen prior to HSCT, knowing that Busulfan-based preparations are less commonly associated with graft failure in young MPSI patients; cell source; and correct donor matching [144,153,154].

### 5.2. Enzyme Replacement Therapy

ERT provides the missing enzyme through weekly administration of the human recombinant IDUA (0.58 mg/kg). It was approved by the Food and Drug Administration (FDA) in 2003 for MPSI and is considered the standard of care for affected patients, especially for the attenuated form of MPSI with no CNS involvement. The enzyme’s uptake from the circulation inside affected cells through a cross-correction mechanism results in the rapid decrease in undegraded GAG accumulation in tissues. This results in the improvement or stabilization of organomegaly, ventricular hypertrophy, and respiratory symptoms, on average. Overall, the greatest benefits of ERT are improved pulmonary function and reduced hepatosplenomegaly, with the best results achieved with early treatment [181,182,183,184,185,186]. Indeed, visceral organs are the main compartments in which the enzyme is delivered, with a little amount that reaches less-vascularized tissues. Depending also on the extent of tissue involvement and age at the time of intervention, some MPSI manifestations, such as ophthalmological, audiological, and cardiopulmonary symptoms of the disease, could be refractory to ERT treatment, with patients having variable responses [165,187,188].

In regard to *dysostosis multiplex*, ERT has been successful in ameliorating general mobility and motility in MPSI patients [155,182,186]. After ERT, many patients demonstrated an improvement in growth and in the 6-min walk, as well as ameliorated joint mobility [189]. After initiation of ERT, several patients showed improved mean height and weight, as well as improved range of motion in shoulders, elbows, and knees [157,181,182]. However, early intervention was a crucial factor in determining the treatment outcome, as ERT was not able to reverse the damage that had already occurred [184,189,190,191]. Joint manifestations do not represent an exception, since ERT shows a limiting effect on cartilage and the growth plate even when treatment is promptly started early in life [192,193]. Indeed, a patient with attenuated MPSI precociously treated with ERT reported preserved skeletal function without development of severe bone alterations, although some joint defects still persisted [184]. Similarly, neonatal ERT performed in MPSI mice did not improve joint disease in regard to inflammation and architecture [194].

Together with other MPSI symptoms, many musculoskeletal manifestations are also refractory to ERT, due to the difficult diffusion of the enzyme into these tissues [165,187,188]. Since the enzymatic blood–brain barrier crossing is also very limited, ERT is not recommended in the case of CNS involvement [165]. However, specific analyses of the risks/benefits of the chosen treatment option are required based on the patients’ phenotypic manifestations [6]. Delivery alternatives have been evaluated for increasing the ERT effect on CNS manifestations, both in terms of different injection route (via cerebrospinal fluid, nasal mucosa) or targeting systems (fusion with monoclonal antibody) (NCT02371226) [195,196].

ERT has also been utilized as a bridge to other therapies to reduce the disease burden, or even a long time after HSCT in order to attenuate persistent disease manifestations [197,198,199,200]. Peri-transplant ERT has been performed with the goal of slowing disease progression right after severe MPSI diagnosis or in between successive transplants, pending a matched donor [27,157,198]. ERT has been also considered after HSCT, with the aim of reducing the residual burden, decreasing life-threatening cardiorespiratory manifestations, and preventing the reoccurrence of some MPSI manifestations months after therapy [201]. No interfering effects have been observed in the case of the combined HSCT and ERT approach, with an improved HSCT outcome and short-term cognitive functions, lower graft-versus-host-disease reports, and attenuated anti-IDUA immunoglobulin G (IgG) formation in patients [198,202].

In regard to the skeletal manifestations of MPSI, improved outcomes were achieved when ERT was combined with HSCT [155]. A case report of combined strategies, tested in a young MPSI patient with a severe spine malformation, showed a great decrease in spinal cord compression and increased growth rate [203]. Continuous treatment with ERT several years after undergoing HSCT improved fatigue and mobility in affected patients [199]. Similarly, ERT performed prior to HSCT resulted in disease stabilization in two monochorionic diamniotic twins, who demonstrated normal linear growth and an improved quality of life, together with a significant amelioration of severe valvular disease and corneal problems [155]. After treatment, their stature overcame typical MPSI percentiles; however, some of the skeletal manifestations were still present, including mild hand stiffness, gibbus, dysrhythmia, neck and teeth problems [155]. Notably, studies performed in adult MPSI mice by Kuehn et al. highlighted how the single treatments did not fully correct the skeletal manifestations of MPSs, with persistently impaired bone remodeling after HSCT and limited effects on bone mass reduction after ERT, with unexpected, increased osteoclastogenesis [123]. By contrast, the combination of the two treatments normalized bone mass, with an effect also in the jaw of treated mice. A patient’s biopsy of the iliac crest showed that the combined therapy caused a decrease in the trabecular bone volume. In addition, surfaces of the osteoblast and osteoclast resulted in the reference range [123].

Even when treatment was successful, many MPSI manifestations in bones, heart, and corneas reappeared after ERT, and additional interventions, including surgical procedures, were required [181,185]. Despite being safe, the treatment itself could be limited by the chronicity of intravenous administrations, infusion-related reactions, the development of specific antibodies, and high costs [182,186,204,205].

### 5.3. Gene Therapy

Because of the therapeutic limitations of standard approaches, alternatives for severe MPSI treatment have been explored. Gene therapy (GT) and genome editing strategies seem to be promising options, especially considering that MPSI is a monogenic disorder and the therapeutic gene can be carried into the patient’s genome. Few clinical applications have been tested in MPSI and MPSs patients, whereas studies on animal models are under investigation in order to further explore these therapeutic options and their effect on this specific disease.

- **In vivo GT.** The vector carrying the therapeutic gene can be directly injected in the case of in vivo GT. Being systemic or targeted to specific tissues, the administration of adeno-associated viruses (AAVs) could induce a long-term target gene expression, with a low genotoxicity risk [206]. The efficacy of this approach for treating MPSI has been demonstrated through many studies performed in animal models, with a better outcome observed when treatment was started early in life [207,208]. In particular, improvements in bone disease, craniofacial parameters, and neurological symptoms were obtained when AAVs were injected into neonatal mice [209]. For treating other MPSs, with the aim to reach bone and cartilage, AAVs have been modified in their capsid region for specifically targeting hydroxyapatite, a binding protein present in bone [210]. In treated mice, prolonged gene expression was observed in this targeted compartment with increased enzyme activity, suggesting that this approach could be translated to other MPSs with skeletal involvement.
  Starting from promising in vitro studies on MPSI fibroblasts, gene therapy using retroviruses (RVs) has been tested in several animal models [211,212,213]. First attempts showed the limited expression stability achieved and the loss over time, highlighting the need for pharmacological immunosuppression to limit the response of cytotoxic T lymphocytes and for gene expression avoidance in antigen-presenting cells [206,214]. When treating adult mice or young dogs with an alpha-1-antitrypsin promoter, many multisystemic manifestations and the cerebral storage were corrected without showing limiting auto-antibody formation [215]. In terms of skeletal defects, GT mice achieved mineralization normalization and a reduction in femur width, whereas no facial dysmorphism was observed in dogs after the early initiation of therapy [116,215].
  Ten years after RV-mediated in vivo GT, lentivirus (LV) efficacy was evaluated first on MPSI fibroblasts, where a long-term gene expression was achieved, and consequently, in animal models, in which even a little improvement in gene correction caused GAG reduction, despite the formation of anti-IDUA IgG limiting the long-term effect [37,216].
- **Ex vivo GT**. In the case of ex vivo GT application, autologous HSCs are employed for carrying the IDUA protein and inducing metabolic correction through the modified progenitors’ engraftment, differentiation, and release of the missing enzyme into local compartments [217]. Overall, after insertion into the genome, supraphysiological *IDUA* expression levels could be achieved, overcoming the ERT limitations of chronic injections. Moreover, an autologous approach, obtained by modifying patient-derived cells, is fundamental for reducing the immunological complications associated with standard HSCT.
  First attempts of ex vivo GT approaches were tested using RVs for transducing donor BM-derived cells and delivering the therapeutic gene in MPSI mice [218]. Visceral enzyme recovery and metabolic reduction were obtained months after treatment, with morphological improvements observed also in brain, but there was a lack of skeletal improvements. When erythroid cells were reprogrammed with targeting LVs, MPSI mice showed long-term enzymatic correction, vacuole reduction in organs, and cognitive improvement [219]; bone defect ameliorations were not investigated. Afterwards, Visigalli et al. demonstrated that results obtained through gene-corrected cells transplanted into adult MPSI mice highly exceeded the HSCT outcome thanks to the supranormal IDUA levels achieved in several tissues [220]. Metabolic and phenotypic defects in hard-to-treat organs were normalized with the amelioration of neurologic manifestations. When considering the skeletal compartment, femur density and dimension analyses, growth plate architecture evaluation, and computed tomography scans of long bones and of the zygomatic arch, values almost comparable to healthy ones were suggested after GT. No significant genotoxicity nor tumorigenic risks were associated with this treatment, as provided by safety preclinical studies that paved the way for the ongoing clinical trial at San Raffaele Hospital (NCT03488394) [221,222]. In this phase 1/2 study, autologous CD34^+^ cells were genetically modified with the LV encoding for the IDUA enzyme and transplanted in eight severe MPSI patients. Overall, this procedure was safe; all patients showed sustained engraftment of gene-corrected cells with blood IDUA activity reaching supraphysiologic levels after GT. Urinary GAG excretion levels reduced to normal or near-normal values by 1-year post-treatment. IDUA activity in the cerebrospinal fluid became detectable by month 3 post-GT in all subjects, accompanied by a progressive decrease in GAG storage. With a median follow-up of 2 years, patients showed stable cognitive and motor performances, reduced joint stiffness, improved or stable findings on brain and spine MRI, and normal growth according to peers (NCT03488394) [222]. These results highlighted the therapeutic potential of ex vivo GT for the treatment of severe MPSI.
- **Gene editing**. A specific gene could be potentially permanently modified for therapeutic purposes through gene editing approaches. The gene of interest could undergo editing, disruption, replacement, or addition in a particular site after double-strand breaks and DNA repair processes. In this context, nucleases are employed for editing the sequence, through the most commonly used zinc-finger nucleases (ZFNs), transcription activator-like endonucleases (TALENs), and CRISPR/Cas9 tools with targeting accuracy [223]. The complementary lacking gene portion, supplied with specific homology arms, could be provided and inserted by the homology direct-repair mechanism in a permanent manner [224]. In the case of a lack of the delivered sequence, non-homologous end-joining could induce a frame shift, with the addition or removal of bases in the proximity of the cut.
  In the case of MPS treatment, the target sequence and CRISPR/Cas9 machinery components were delivered through a liposome formulation into newborn MPSI mice; it allowed metabolic correction in some compartments, including in the cardiac tissue, with no CNS effect nor evaluation of bone amelioration [225]. Genome-edited human HSCs, modified through the CRISPR/Cas9 technology, were transplanted in adult immunodeficient MPSI mice and caused a limited increase in circulating IDUA, that was, however, enough for causing metabolic normalization of the main visceral organs and partial improvements in the CNS manifestations. This approach corrected the phenotypic bone alterations of treated mice, normalizing skull and long-bone thickness [226].
  On the other hand, novel genome editing strategies use delivered ZFN for inserting the therapeutic gene and inducing normal transgene expression. By targeting the albumin locus, positive preliminary preclinical results in visceral and neurological manifestations paved the way for its application in MPSI-attenuated patients. Since 2016, Sangamo Therapeutics started an ongoing phase 1/2 clinical study that shows a lack of severe side effects, but low gene expression (NCT02702115; NCT04628871) [227]. No indications about its effect on the skeletal outcome were reported up to now.
- **Non-viral delivery methods**. Together with AAV-, RV-, and LV-based delivery of the therapeutic gene, non-viral methods have been applied for the treatment of MPSI. By using a hydrodynamic technique for targeting the liver and transposing the cassette of interest into the genome, the Sleeping Beauty (SB) transposon system represents one of the most applied non-viral approaches for delivering the therapeutic transgene. Its application for MPSI treatment has demonstrated an effect in reducing biochemical and metabolic defects in mice [228]. In terms of skeletal defects, SB treatment caused a reduction in zygomatic thickness, with no growth plate defects observed in long bones and a loss of swollen cells in the BM [228]. No improvements in cortical thickness or mid-diaphyseal bone regions’ dimensions were reported.

Despite these safer approaches, concerns about GT for MPSI treatment remain; a high price and limited availability, the immune system triggering against the delivery particle, genotoxicity, and uncertain feasibility in MPSI patients could represent the main limitations for its application, and further tests are needed for its applicability in patients [229,230].

### 5.4. Potential Alternative Approaches for MPSI Treatment (Table 1)

- **Storage reduction strategies**. More precise studies of the pathophysiology behind MPS disorders have encouraged the investigation of several small-molecule therapies. In particular, storage reduction approaches act on blocking GAG production, instead of promoting their catabolism, by balancing synthesis over degradation. Overall, in contrast to ERT, the idea is to avoid GAG synthesis and disease worsening, and a therapeutic benefit for skeletal manifestations could also be possible [231,232].
  Genistein (5,7-dihydroxy-3-[4-hydroxyphenyl]-4H-1-benzopyran-4-one) is an isoflavone that binds and inactivates the epidermal growth factor receptor, which is fundamental for GAG formation [233]. When tested in MPSII and III patients (PO 5 mg/kg/day), improvements in some clinical and behavioral aspects were observed, likely due to Genistein’s ability to cross the blood–brain barrier [234,235]. Despite articular mobility that resulted in being ameliorated when administered, further clinical efficacy is still to be determined, especially considering side effects observed in treated mice that are probably due to a broader effect of the molecule on different pathways [234,235,236].
  Alternative strategies include the administration of small compounds for reducing secondary molecules, such as gangliosides, whose synthesis could be blocked using Miglustat [237]. On the other hand, short interfering RNA (shRNA) molecules could be applied for silencing genes that encode for enzymes that take part in GAG metabolism [238]. This approach was tested in MPSI fibroblasts and caused reduced synthesis, highlighting its possible application for MPS treatment.
- **Nonsense suppression treatment**. In MPSI patients with a genotype characterized by the presence of nonsense pathogenic variants in the *IDUA* gene, nonsense suppression therapies could be used. Specific molecules could promote the bypassing of the termination codon, allowing elongation to proceed, and subsequently, allow for the production of a full-length protein that retains its enzymatic properties. Additionally, a partial enzyme restoration could significantly ameliorate disease manifestations, considering MPSI as a promising candidate. The combination of nonsense-mediated mRNA decay attenuation and suppression treatments, such as gentamicin or NB84, was tested in MPSI mice, resulting in beneficial effects without toxicity, restoring the enzymatic activity, and reducing GAG accumulation [239]. The progression of skeletal involvement, together with cardiac and cerebral hard-to-treat compartments, was delayed after long-term treatment with NB84 in MPSI animals, as demonstrated by both improved architecture parameters and osteoclast number reduction [240]. These promising results promoted these alternative applications for treating MPS patients, although toxicity remains a limiting factor.
- **Chaperone therapy**. Chaperones could represent a valid treatment option in case of mutations that impair protein folding and consequent trafficking and accumulation. This therapy stabilizes these misfolded proteins by mainly rearranging their hydrophobic portion, and avoids storage and degradation, since enzyme activity and trafficking have been corrected [241]. Differently from ERT, the CNS and several organs could be reached, along with no immunogenicity and administration ease. Nonetheless, despite the application for many MPSs, a restriction to specific mutations, off-target toxicity, and enzyme inhibition risk could limit its applicability [241,242].
- **Others**. New strategies for treating MPSs are currently under investigation. Some preliminary in vitro results have been obtained after overexpression of the *TFEB* (transcription factor EB) gene, which is important for the autophagy regulation that is severely affected in MPSs. Altering the autophagic-lysosomal pathway in MPSIIIA resulted in the clearance of GAG accumulation [243].

Alternatively, the recombinant protein NK1, which is able to bind the extracellular excess of HS, was tested in MPSI as well as other MPS fibroblasts, and showed improvement in in vitro GAG content. This approach suggested that competitive strategies could be applied for reducing excessive GAG storage in tissues and restoring affected signaling pathways [37].

Furthermore, therapies involving secondary pathways represent appealing strategies for treating MPSI in order to target disease manifestations with a low response to standard treatments.

### 5.5. Alternative Treatments Focused on Skeletal Defects

Despite several treatment options that are available for MPSI, there is a need for new therapeutic approaches that specifically target skeletal disease in affected patients. Strategies involving anti-inflammatory drugs, cytokine use, growth factor therapies, and specific targeting approaches are here described:

- **Anti-inflammatory drugs.** Since inflammation highly regulates MPSI pathogenesis and inflammatory pathways are activated in affected patients, targeting inflammation with anti-inflammatory drugs could limit its effects on connective tissue alterations and disease progression.
  Pentosan polysulfate (PPS), a molecule with prochondral activity and anti-inflammatory properties, was tested in the canine model of MPSI and noted to cause an overall decrease in GAG accumulation [252]. Similarly, adult MPS patients that received the PPS therapy combined with the standard ERT showed improvements in hip, knee, and ankle mobility, suggesting that inflammatory impairment plays a crucial role in MPSI [253]. Alternatively, infliximab, with its tumor necrosis factor (TNF)α-binding properties and effect on the TLR4–TNFα axis, was injected in MPS animals, and therapeutic benefits were appreciated when combined with standard ERT [254].
  Additionally, the TNF inhibitor adalimumab is currently under investigation in a phase 1/2 study; its effect on the *dysostosis multiplex* manifestations is being tested in MPSI patients, together with safety and efficacy analyses (NCT03153319).
  Combinational approaches to standard therapies could substitute ERT and avoid antibody formation in the case of early treatment after newborn screening, with the final goal of treating younger patients [252].
- The use of **MMPs** and **apoptosis inhibitors, cytokines, and WNT/β-catenin agonists** could represent an alternative approach for counteracting the bone defects observed in many MPSs [48]. In particular, Simonaro et al. demonstrated that matrix metalloproteinases MPP2 and MMP9 were impaired, causing matrix and bone formation alterations, and that reduced mineralization resulted from defects in hypertrophic chondrocytes. In addition, the high level of cartilage death was compensated for by TGFβ release to increase the proliferation rate. Cytokines and MPPs highly moderate the pathways involved in skeletal balance, and targeting these key players could be a valuable therapeutic approach to be combined with standard treatments, with the aim of achieving the correct bone formation and maturation, especially in young patients.
- **Growth factor therapy**. GAG accumulation severely impairs the bone milieu and the released growth factors’ role, contributing to MPSI skeletal degeneration. By knowing the specific interaction and function of the GF in the context of bone and other organs, targeting specific receptors could be an alternative strategy to couple with standard approaches [125]. A combined approach using C-type natriuretic peptide and ERT, tested in MPSVII mice for promoting growth improvement, resulted in a synergic effect that overcame the two monotherapies; regulating chondrocyte apoptosis on one side and thickening of the growth plate on the other caused amelioration of the hypertrophic zone composition and, overall, articular cartilage growth, suggesting that the skeletal defects of MPSs could benefit from this combined strategy [255].
  Recent studies have highlighted the fine regulation of the mammalian target of rapamycin complex 1 (**mTORC1**) signaling for appropriate longitudinal skeletal growth; in particular, impaired collagen trafficking in osteoclasts was hypothesized in the case of MPS mice, with delayed secretion due to unbalanced collagen metabolism. Altered TORC1-dependent post-translational modifications in UV radiation resistance-associated (UVRAG) protein were observed in patient-derived chondrocytes and precursors, which were isolated from MPSVI and MPSI primary samples, respectively. Bone formation was restored after a reduction in mTORC1 signaling or Tat-Beclin1, a peptide able to induce autophagy, overall improving the collagen composition in cartilages in MPSVI and MPSVII mice [256].
- Abnormal **osteoactivin** expression was found in some MPSs. Osteoactivin has the fundamental role of regulating endochondral ossification, proper osteoblast differentiation, and autophagy. Acting on this pathway could represent a specific therapeutic approach for ameliorating the bone involvement in MPSI patients [140].
- **MSCs** represent potential therapeutic targets because of their role in immunomodulation, regeneration, and osteoblast or chondrocyte differentiation, especially when considering combined approaches for MPSI. Positive effects on motor coordination were observed when healthy BM-derived multipotent stem cells were intracerebroventricularly injected in affected mice [257]. Modified MSCs were successfully able to normalize enzyme activity and retinal defects in MPS mice when precociously treated, highlighting the feasibility of neonatal ex vivo GT [258]. When injected in patients with severe MPSI, bone mineral density was maintained or slightly improved, probably due to the already set skeletal defects and late time of intervention [259]. The MSC effect on precociously halting *dysostosis multiplex* progression needs to be evaluated.
- **Biphosphonates** have been considered as a supportive therapy for MPSs in light of their ability to counteract bone resorption by impairing several osteoclast activities, promoting osteoclast apoptosis, and increasing bone mass [260]. In particular, neridronate given intravenously has been reported to improve functional bone performance and the radiological phenotype of a patient affected by MPSIVA [261].

### 5.6. Early Treatment of MPSI Disease Manifestations

Due to the progressive nature of MPSI, early diagnosis and the prompt initiation of treatment are crucial in order to prevent irreversible organ damage. Once GAG deposition has occurred, it cannot be reversed through available treatment strategies. Kigma et al. speculated that the skeletal growth of affected mice was not influenced by the FGF2 effect, which is different from healthy bones because of GAG accumulation leading to impaired growth factor distribution [125]. External GAGs also impaired the bone milieu and the function of released growth factors, thus contributing to the skeletal degeneration characteristic of MPSI. 

Clinical experience with MPSI siblings’ cases highlighted that early treatment in affected siblings of a previously identified proband led to improved overall morbidity and cognitive outcome [184,262]. Furthermore, many available treatments have demonstrated their increased efficacy when started early in life, identifying age at treatment as a predictive factor for a better clinical outcome [156,158,165,183,184,185,194,263,264]. In particular, both experiences in animal models and MPSI patients showed an improvement in bone defects in cases of early treatments, considering ERT [184] and/or HSCT [265,266,267]. In view of this, a neonatal GT approach could be helpful because it allows for a very early treatment and, in parallel, it is associated with a reduced risk of immunological complications (i.e., immune response against the therapeutic enzyme). This effect is especially apparent in cases of CNS involvement, as observed in many studies on MPS animal models performed over the years with cognitive improvement or increased enzymatic activity [207,263,268,269]. When considering in utero approaches, Bose et al. demonstrated that a base editor with AAV delivery was responsible for the amelioration of cortical portions of skull and long bones, facial coarsening, and also lordosis of MPSI mice, together with an improvement in visceral organ involvement, partial cognitive recovery, and antibody formation only in the postnatal phase [270]. Similarly, positive results have been obtained in terms of symptom onset delay in MPSVIII mice after RV treatment and both cerebral and bone defects ameliorations when in utero transplantation was performed in MPSVII mice [271,272]. Promising early approaches could improve the treatment outcome in cased of diseases with late therapies; however, further safety analyses need to be performed prior to clinical translation.

Overall, the best treatments or combination of therapies should be considered based on the safety profile, invasiveness, chronicity, and overall outcomes. Nonetheless, if diagnosis and the start of treatment are performed prior to disease manifestation onset and proper phenotype classification, difficulties in therapeutic decision making could occur. Precise markers associated with clinical severity that could be used in the first months of life, before phenotypic changes manifest, need to be identified and could be helpful for further evaluations [36,273].

## 6. Conclusions

The underlying pathological and molecular mechanisms responsible for the skeletal phenotype in MPSs remain not fully understood. Therefore, current standard therapeutic approaches have limited benefits. HSCT and/or ERT have been shown to improve joint mobility in affected patients, although the outcome is largely influenced by the age at which treatment is initiated, and residual disease burden remains in the long term. Alternative therapeutic approaches, including gene therapy and editing, are currently under preclinical and/or clinical investigation.

A better understanding of the mechanisms that contribute to skeletal defects in MPSs could help in developing novel therapeutic strategies that directly target the skeletal phenotype in affected patients, allowing us to not only prevent skeletal manifestations, but also to potentially reverse them. Overall, an improvement in MPSI patients’ quality of life could be achieved through early diagnosis, identification of disease severity markers, and prompt initiation of treatment with targeted therapies.

## Figures and Tables

**Table 1:** Clinical trials with musculoskeletal investigations. List of recruiting (green), active and not recruiting (orange), not yet recruiting (blue), and terminated (gray) clinical trials from 2000 to 2022, with detailed planned analyses on bone manifestations. * Estimated patients’ enrollment, NA not available.

Identifier (Phase/Type)	Date	Title	Sponsor Name	Age (Enrolled Patients)	Musculoskeletal Investigation	Results
NCT00912925 (3)	2000–2001	Clinical Study of Aldurazyme in Patients With Mucopolysaccharidosis (MPS) I	Genzyme (Sanofi Company)	≥5 years (45)	6-min walk test distance, active joint range of motion (+26 weeks)	[244]
NCT00146770 (3)	2001–2005	Phase 3 Extension Study of the Safety and Efficacy of Aldurazyme^®^ (Laronidase) in Mucopolysaccharidosis I (MPS I) Patients	Genzyme (Sanofi Company)	Child, adult (45)	6-min walk test distance, active joint range of motion (+182 weeks)	[244]
NCT00146757 (2)	2002–2005	A Study Evaluating the Safety and Pharmacokinetics of Aldurazyme^®^ (Laronidase) in MPS I Patients Less Than 5 Years Old	Genzyme (Sanofi Company)	≤5 years (20)	Measure of standing height/lying-length-for-age (+52 weeks)	[245]
NCT00144794 (observational)	2003-	Mucopolysaccharidosis I (MPS I) Registry	Genzyme (Sanofi Company)	Child, adult (1500 *)	Description of variability, progression, and natural history of MPS I	[34,246,247]
NCT00144781 (4)	2004–2006	A Dose-optimization Study of Aldurazyme^®^ (Laronidase) in Patients With Mucopolysaccharidosis I (MPS I) Disease	Genzyme (Sanofi Company)	Child, adult (34)	6-min walk test distance (+26 weeks)	[248]
NCT01521429 (observational)	2009–2019	Longitudinal Study of Bone Disease in Children With Mucopolysaccharidoses (MPS) I, II, and VI	Lundquist Institute for Biomedical Innovation at Harbor-UCLA Medical Center	5–35 years (55)	Analyses of bone density, geometry, strength and muscle fat, bone turnover, growth measurements (+1–2–3 years)	NA
NCT00418821 (4)	2010-	A Study of the Effect of Aldurazyme^®^ (Laronidase) Treatment on Lactation in Female Patients With Mucopolysaccharidosis I (MPS I) and Their Breastfed Infants	Genzyme (Sanofi Company)	Child, adult (2 *)	Drug effect in infants in terms of growth and development	[249]
NCT01173016 (1)	2012–2016	Administration of IV Laronidase Post Bone Marrow Transplant in Hurler	Masonic Cancer Center, University of Minnesota	5–13 years (11)	Analyses in growth velocity, muscle strength, joint range of motion, 6-min walk test distance (+24 months)	[250]
NCT02067650 (observational)	2013–2016	Ultrasound Findings of Finger, Wrist and Knee Joints in Mucopolysaccharidosis	Children’s Hospital of Eastern Ontario	2–99 years (18)	Finger, wrist, and knee joint abnormalities, synovitis/tenosynovitis	NA
NCT02171104 (2)	2014-	MT2013-31: Allo-HCT for Metabolic Disorders and Severe Osteopetrosis	Masonic Cancer Center, University of Minnesota	≤55 years (100 *)	Incidence of radiographic aspects of the disease (+1–2 years)	NA
NCT02437253 (1/2)	2015–2017	Effects of Adalimumab in Mucopolysaccharidosis Types I, II and VI	Lundquist Institute for Biomedical Innovation at Harbor-UCLA Medical Center	≥5 years (2)	Height and weight, physical function, joint range of motion, 6-min walk test distance, and strength testing, serum joint inflammation (+16–32 weeks)	[251]
NCT03053089 (1/2)	2015–2018	Safety and Dose Ranging Study of Human Insulin Receptor MAb-IDUA Fusion Protein in Adults and Children With MPS I	ArmaGen, Inc.	≥2 years (21)	Functional capacity in terms of 6-min walk test distance, shoulder range of motion (+26 weeks)	[196]
NCT03153319 (1/2)	2017-	Study to Evaluate the Safety and Efficacy of Adalimumab in MPS I, II, and VI	Lundquist Institute for Biomedical Innovation at Harbor-UCLA Medical Center	≥5 years (14 *)	Joint range of motion (+16–32 weeks)	NA
NCT02298712 (observational)	2018-	Biomarker for Hurler Disease (BioHurler)	CENTOGENE GmbH Rostock	≥2 months (1000 *)	PB markers for early diagnosis and avoidance of musculoskeletal manifestation appearance	NA
NCT03488394 (1/2)	2018-	Gene Therapy With Modified Autologous Hematopoietic Stem Cells for the Treatment of Patients With Mucopolysaccharidosis Type I, Hurler Variant (TigetT10_MPSIH)	IRCCS San Raffaele	≤11 years (8)	Growth, range of motion, clinical and radiological evaluations (+1–3–5 years)	[222]
NCT04227600 (1/2)	2020-	A Study of JR-171 in Patients With Mucopolysaccharidosis I	JCR Pharmaceuticals Co., Ltd.	Child, adult (19 *)	6-min walk test distance (+13 weeks)	NA
NCT04453085 (1/2)	2021-	An Extension Study of JR-171-101 Study in Patients With MPS I	JCR Pharmaceuticals Co., Ltd.	Child, adult (15 *)	6-min walk test distance	NA
NCT04958070 (observational)	2021-	The Intensively Follow-up Examinations for Asymptomatic MPS I Infants in Taiwan	Mackay Memorial Hospital	3–8 years (median, 16)	Regular physical examinations	NA
NCT04532047 (1)	2021-	In Utero Enzyme Replacement Therapy for Lysosomal Storage Diseases (IUERT)	University of California, San Francisco	18–50 years (10 *)	Growth, mobility, skeletal survey evaluations (+6 years)	NA
NCT04284254 (1/2)	2022-	MT2018-18: Sleeping Beauty Transposon-Engineered Plasmablasts for Hurler Syndrome Post-Allo-HSCT	Masonic Cancer Center, University of Minnesota	3–8 years (36 *)	Growth velocity in terms of cm/year, sitting and standing height (+1 year)	NA

## Data Availability

No new data were created in this study. Data sharing is not applicable to this article.
